# Short-Chain Modified SiO_2_ with High Absorption of Organic PCM for Thermal Protection

**DOI:** 10.3390/nano9040657

**Published:** 2019-04-25

**Authors:** Fuxian Wang, Shiyuan Gao, Jiachuan Pan, Xiaomei Li, Jian Liu

**Affiliations:** 1 Guangdong Provincial Key Laboratory of Emergency Test for Dangerous Chemicals, Guangdong Institute of Analysis, Guangzhou 510070, China; wangfuxian@fenxi.com.cn (F.W.); panjiachuan@fenxi.com.cn (J.P.); 2 The Engineering Research Center of None-Food Biomass Efficient Pyrolysis and Utilization Technology of Guangdong Higher Education Institutes, Dongguan University of Technology, Dongguan 523808, China; gsy0113@126.com (S.G.); L13728356646@163.com (X.L.); 3 Guangdong Provincial Key Laboratory of Distributed Energy Systems, School of Chemical Engineering and Energy Technology, Dongguan University of Technology, Dongguan 523808, China

**Keywords:** thermal protection, organic PCM, SiO_2_ aerogel, short-chain modification, PCM absorption

## Abstract

Organic phase change materials (PCMs) have great potential in thermal protection applications but they suffer from high volumetric change and easy leakage, which require “leak-proof” packaging materials with low thermal conductivity. Herein, we successfully modify SiO_2_ through a simple 2-step method consisting of n-hexane activation followed by short-chain alkane silanization. The modified SiO_2_ (M-SiO_2_) exhibits superior hydrophobic property while maintaining the intrinsic high porosity of SiO_2_. The surface modification significantly improves the absorption rate of RT60 in SiO_2_ by 38%. The M-SiO_2_/RT60 composite shows high latent heat of 180 J·g^−1^, low thermal conductivity of 0.178 W·m^−1^·K^−1^, and great heat capacity behavior in a high-power thermal circuit with low penetrated heating flow. Our results provide a simple approach for preparing hydrophobic SiO_2_ with high absorption of organic PCM for thermal protection applications.

## 1. Introduction

Phase change materials (PCMs), a kind of materials with significant amounts of enthalpy through their phase transformations from liquid to solid [1,2], have attracted attention for their application in the thermal protection [3,4], energy storage [5,6,7], thermoregulation [8,9] and energy saving [10]. In thermal protection, the PCMs usually work as heat capacitors since they could absorb heating/cooling thermal energy in the thermal circuit to protect the back-end equipment/materials from thermal runaway/overcooling. Among different PCMs, organic PCMs possess the merits of good chemical stability, low supercooling, high latent heat, and they are generally non-toxic [11]. They have been widely used in thermal packaging industry to maintain temperature sensitive products within the required temperature range during their transfer [12]. However, the application of organic PCMs in thermal protection is largely hindered by the high volumetric change and easy leakage at the liquid state [13]. Therefore, providing “leak-proof” packaging is one of the biggest challenges for the application of organic PCM in thermal protection.

Various packaging materials have been tested to encapsulate organic PCM to form phase change composites. Prepolymers such as Melamine-formaldehyde (MF) resin [14,15], urea-formaldehyde (UF) resin [16], poly(urea−urethane) (PU) resin [17,18], gelatin and Arabic gum [19], and polymethylmethacrylate (PMMA) [20] can encase the organic PCMs via sol-gel, electrostatic spinning and microfluidics methods. These phase change composites usually turn into micro/nano encapsulations or phase change fibers. However, they suffer low phase change enthalpy, complex preparation process, and high cost. The other way is to use porous materials to absorb the organic PCMs by capillary force and surface tension. Porous carbon-based materials such as expanded graphite [21,22], graphene form [23,24], carbon nanotube sponge [25,26,27,28] could encase organic PCMs, but the high conductivity of carbon-based materials makes them unfavorable for thermal protection application. Moreover, carbon materials like graphene form, carbon nanotube sponge are currently too expansive for industrial application. Porous oxide materials such as bentonite [29], diatomite [30], and expanded perlite [31] can absorb PCMs to form phase change composite with low thermal conductivity, but these phase change composites are seriously restricted by their poor absorption of organic PCMs.

Silica (SiO_2_) aerogel, a scaffold ultralight material with high pore volume, large surface area, low thermal conductivity and high thermal stability, is a promising material for encapsulating organic PCMs [32,33] for thermal protection applications. However, the SiO_2_ aerogel is hydrophilic with hydroxyl on the surface, whereas organic PCMs are long chain alkane with hydrophobic property [34]. This incompatibility significantly restricts the mass fraction of organic PCMs in the SiO_2_ aerogel. Efforts have been paid to modify the SiO_2_ surface to be hydrophobic by inducing organic groups. Specifically, amine/methyltrimethoxysilane and methyltrimethoxysilane-dimethyldimethoxysilane modifications have been reported to effectively change the hydrophilicity of SiO_2_ [35,36,37]. However, the long carbon chains of the induced organic groups inevitably result in significant narrowing of the pore size of SiO_2_ aerogel, and consequently a decrease in absorption capacity of organic PCMs [38,39,40]. Therefore, developing hydrophobic SiO_2_ with high porosity is the crucial task ahead for applying SiO_2_ as an encapsulating material for organic PCMs composite. Actually, Rao et al. have demonstrated the possibility of using short-chain -Si-(CH_3_)_3_ modification to prepare hydrophobic SiO_2_, but the 8-step synthesis is rather complicated, and they did not investigate its application for organic PCM absorption [41]. Nevertheless, this inspires us to consider short-chain modified SiO_2_ as organic PCM packing material. Short-chain -Si-(CH_3_)_3_ occupies less space itself, and thus short-chain modification is expected to retain more porosity of SiO_2_ compared to the long-chain modification mentioned above. Therefore, our efforts have been applied to simplify the 8-step synthesis of hydrophobic SiO_2_ developed by Rao et al., evaluate its absorption capacity of organic PCM, and promote its application for thermal protection.

In this work, a simple 2-step method was used to prepare hydrophobic SiO_2_. N-hexane was found to effectively activate the surface of SiO_2_, which further benefits the replacement of hydroxy with short-chain silicane via hexamethyl disilazane silanization. The short-chain modified SiO_2_ (M-SiO_2_) exhibits excellent hydrophobic property while maintaining the intrinsic high porosity of SiO_2_. The M-SiO_2_ shows much-improved absorption of RT60 (a typical organic PCM) than SiO_2_. Compared to SiO_2_-based composite, the M-SiO_2_-based phase change composite shows improved thermal protection behavior and greater latent heat. Moreover, the M-SiO_2_-based phase change composite shows good reversible stability and no leakage of PCMs was observed after 200 heating-cooling cycles. The M-SiO_2_ aerogel-based phase change composite could rectify the large ambient heat flow and exhibits great potential for thermal protection applications.

## 2. Materials and Methods

### 2.1. Materials

The hydrophilic SiO_2_ aerogel and technical grade paraffin RT60 (melting point Tm = 60 °C) were purchased from Guangzhou GBS Technology Co, Ltd, Guangzhou, China. and Hangzhou ruhr energy science and technology Co, Ltd, Hangzhou, China, respectively. Hexamethyl disilazane and n-hexane were provided by Aladdin Co, Ltd, Shanghai, China.

### 2.2. Preparation of M-SiO_2_ Aerogel and RT60/M-SiO_2_ Composite

**Figure 1:**
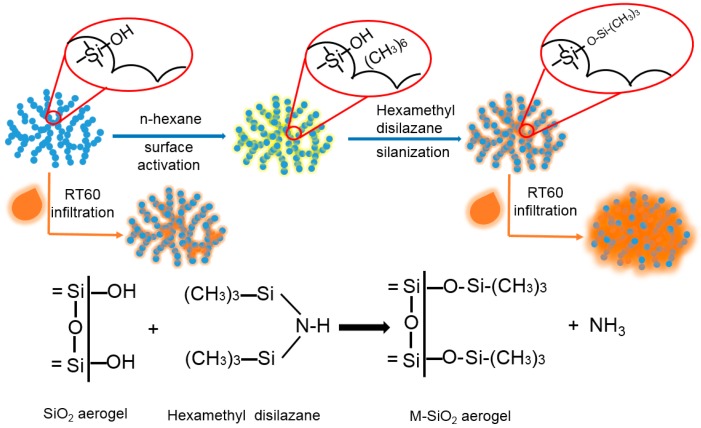
Illustration of M-SiO_2_ aerogel and RT60/M-SiO_2_ aerogel preparations.

The M-SiO_2_ aerogel was synthesized by the method reported previously with minor modification [41], as shown in Figure 1. First, 20 g of hydrophilic SiO_2_ aerogel was dried at 80 °C for 12 h, then poured into the 200 mL of n-hexane to activate the surface of SiO_2_ aerogel. After ultrasonic dispersion at 50 °C for 1 h, the activated SiO_2_ aerogel was centrifuged from the suspension and immersed into the 200 mL Hexamethyl disilazane for 48 h to replace the hydroxy with silicane and release ammonia at room temperature. Last, the modified SiO_2_ aerogel was centrifuged and dried under vacuum at 50 °C for 5 h. The hydrophobic SiO_2_ aerogel was obtained and defined as modified SiO_2_ aerogel (M-SiO_2_ aerogel). For comparison, Hexamethyl disilazane silanized SiO_2_ without n-hexane activation was also prepared and defined as unactivated modified SiO_2_ (unactivated M-SiO_2_). The PCM composite was prepared through the infiltrating method. 80 g of RT60 was kept in a thermal chamber at 80 °C until it completely melted. Then 20 g of M-SiO_2_ aerogel was added into the liquid RT60 to encapsulate the melting RT60. After stirring at 80 °C for 2 h, the RT60/M-SiO_2_ aerogel was fabricated.

### 2.3. Characterization of M-SiO_2_ Aerogel and RT60/M-SiO_2_ Aerogel

The morphology and microstructure of SiO_2_ aerogel and M-SiO_2_ aerogel were observed by a field emission scanning electron microscopy (SEM SU8020, Hitachi, Tokyo, Japan). The water contact angle of the SiO_2_ aerogel and M-SiO_2_ aerogel were observed by a water contact angle meter (SDC 500, Dongguan Chengding Technology Co. Ltd, Dongguan, China.). The SiO_2_ aerogel and M-SiO_2_ aerogel were compressed into blocks, then pure water was dropped onto the surface of SiO_2_ aerogel and M-SiO_2_ aerogel blocks. The microscopic pores of SiO_2_ aerogel and M-SiO_2_ aerogel were observed by a transmission electron microscopy (FEI Tecnai G20, Hillsboro, OR, USA). The structure of the phase change material was characterized by FT-IR spectra and XRD. The FT-IR spectra were recorded on a Bruker 550 from 400–4000 cm^−1^ using KBr pellets. The XRD pattern was scanned from 10–70° with intervals of 0.2°. The phase change temperature and latent heat of SiO_2_ aerogel and M-SiO_2_ aerogel-based phase change materials were measured using a differential scanning calorimeter (Q200, TA). For DSC measurements, 5–8 mg of each sample was sealed in an aluminum pan. The heating rate is 10 °C·min^−1^ and the N_2_ flow rate was 50 mL·min^−1^. The thermal stability of SiO_2_ aerogel and M-SiO_2_ aerogel-based phase change composite was investigated by the thermogravimetric analysis (TGA) using a thermal analyzer (Q600 SDT, TA, URT100, New Castle, PA, USA). The measurements were conducted by heating the samples from room temperature to 600 °C at a heating rate of 10 °C·min^−1^ under N_2_ atmosphere with a flow rate of 100 mL·min^−1^. The thermal conductivity of SiO_2_ aerogel and M-SiO_2_ aerogel-based phase change composite was measured using a thermal constants analyzer (Hot Disk TPS 2500 S, Hot Disk AB, Gothenburg, Sweden). The thermal insulation performance of SiO_2_ aerogel and M-SiO_2_ aerogel-based phase change material was conducted in a double layer cubic box. The SiO_2_ aerogel and M-SiO_2_ aerogel-based phase change material was placed in the inner face of the box. The box was heated to 65 °C and placed in a cool stage at 20 °C. Then, the temperature of the inner box was measured by thermocouples. For comparison, the XPS foam was also used as thermal insulation material, and the synergistic effect of XPS foam, SiO_2_ aerogel and M-SiO_2_ aerogel-based phase change composite was also investigated. The temperature data was recorded by Agilent 34970A, Agilent Technologies Inc, Santa Clara, CA, USA.

## 3. Results and Discussion

### 3.1. FT-IR Investigation of SiO_2_, Activated SiO_2_ and M-SiO_2_ Aerogels

**Figure 2:**
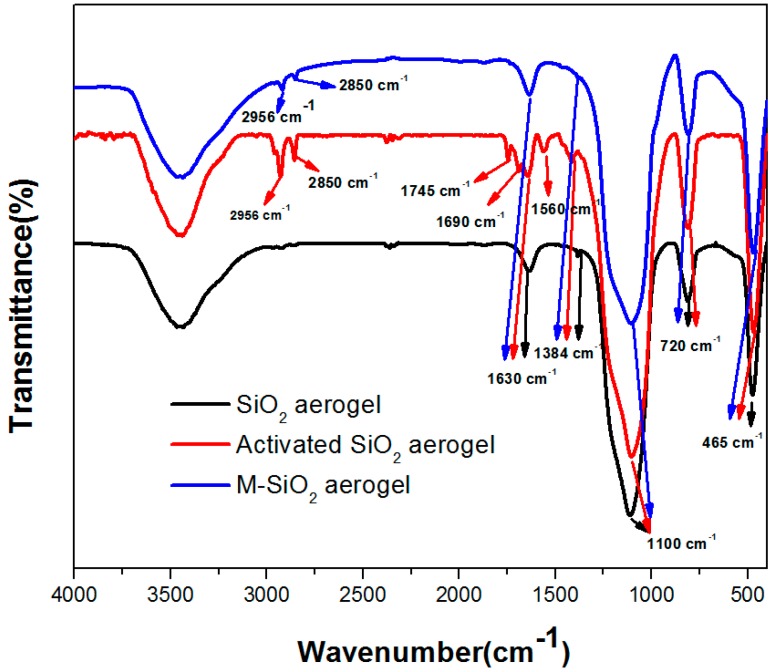
The FT-IR spectrum of SiO_2_, activated SiO_2_, and M-SiO_2_ aerogels.

To analyze the structures of SiO_2_ aerogel and M-SiO_2_ aerogel-based PCM, the FT-IR analysis was conducted. The FT-IR spectra of SiO_2_ aerogel, activated SiO_2_ aerogel, and M-SiO_2_ aerogel are displayed in Figure 2. The low intensity bands at 3000–3700 cm^−1^ and around 1630 cm^−1^ are assigned to -OH stretching vibrations [42], the peaks at 1100, 788, and 465 cm^−1^ are ascribed to the bending vibration of Si-O-Si. The activated SiO_2_ aerogel shows two absorptive peaks at 2956 cm^−1^ and 2850 cm^−1^, which correspond to the stretching vibration of -CH_3_ and -CH_2_-. In addition, the activated SiO_2_ show peaks at 1745 cm^−1^ and 1690 cm^−1^, which could be assigned to the C=O stretching vibration [43], yet the mechanism of the C=O formation in the activated SiO_2_ is not clear. The band at 1560 cm^−1^ can be attributed to the stretching vibration of C=C [44]. The M-SiO_2_ aerogel shows two weak absorptive peaks at 2956 cm^−1^ and 2850 cm^−1^, verifying the successful surface modification. The FT-IR spectrum of other samples can be found in Figure S1. Specifically, in RT60, the peaks at 2956 cm^−1^ and 2850 cm^−1^ correspond to the stretching vibration of -CH_3_ and -CH_2_-, the peaks at around 1465 cm^−1^ belong to the deformation vibration of -CH_2_ and -CH_3_, and the peak at 1415 cm^−1^, 1384 cm^−1^ and 720 cm^−1^ are due to the CH vibration [45], CH_3_ deformation [46] and in-plane rocking vibration of -CH_2_ respectively [19]. The spectrum of the RT60/SiO_2_ aerogel and RT60/M-SiO_2_ aerogel keeps all of the absorptive peaks of RT60, SiO_2_ aerogel or M-SiO_2_ aerogel, indicating that the combination of the RT60 and SiO_2_ aerogel or M-SiO_2_ aerogel is a physical process.

### 3.2. Wettability of SiO_2_ and M-SiO_2_ Aerogels

**Figure 3:**
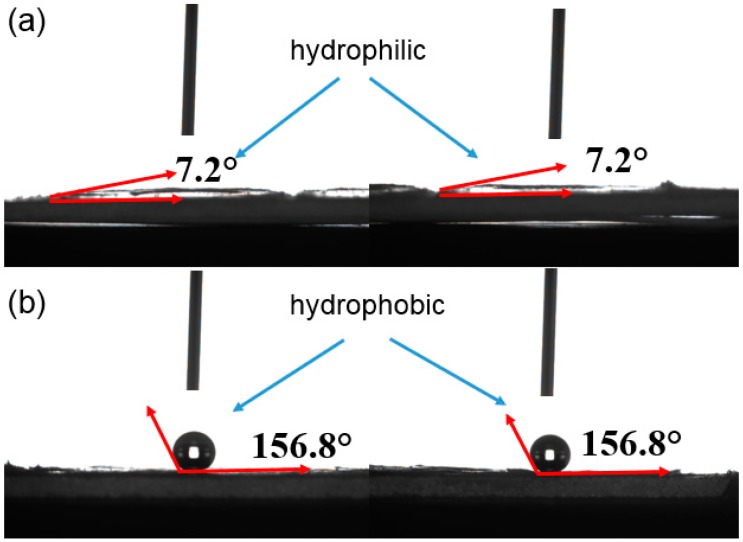
Water contact angles of SiO_2_ aerogel (**a**) and M-SiO_2_ aerogel (**b**).

The water contact angle of the SiO_2_ aerogel and M-SiO_2_ aerogel is illustrated in Figure 3. The water contact angle of the SiO_2_ aerogel is 7.2° (Figure 3a), suggesting that the SiO_2_ aerogel is hydrophilic, which is caused by the surface group of –Si-OH. In contrast, Figure 3b shows that the water contact angle of M-SiO_2_ aerogel is 156.8°, indicating the successful hydrophobic modification. The obtained M-SiO_2_ aerogel is hydrophobic, which is consistent with previous reports, where the M-SiO_2_ aerogel was used as a super-hydrophobic layer for light transition systems [47].

### 3.3. Porosity and Absorption Capacity of SiO_2_, Unactivated M-SiO_2_, and Activated M-SiO_2_ Aerogels

**Figure 4:**
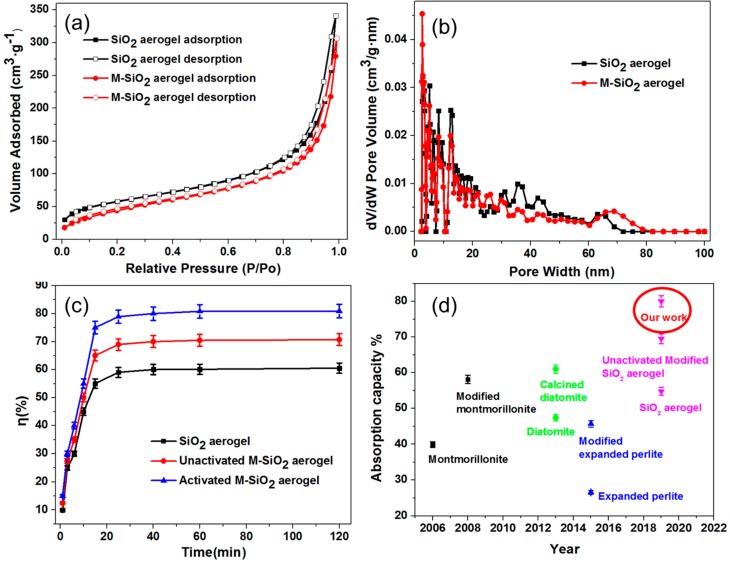
N_2_ adsorption/desorption isotherms of SiO_2_ and M-SiO_2_ aerogels (**a**), pore size distribution of SiO_2_ and M-SiO_2_ aerogels (**b**), and absorption capacities of SiO_2_ and M-SiO_2_ aerogels: Our results (**c**) and a summary of the recent studies (**d**) are shown.

To evaluate the porosity of SiO_2_ and M-SiO_2_ aerogels, the N_2_ adsorption and desorption isotherms were measured. As shown in Figure 4a, the N_2_ adsorption/desorption curve shows a steep hysteresis loop [48], indicating the week connection between N_2_ and the SiO_2_ aerogel. This is because the mesoporous was built within SiO_2_ or M-SiO_2_ particles by Van der Waals’ force. The sharp rise at a relative pressure (P/P0) of ~0.9 indicates the existence of mesoporous with narrow pore size. The BET surface area (SBET) of SiO_2_ aerogel and M-SiO_2_ aerogel is 350 m^2^·g^−1^ and 306 m^2^·g^−1^, suggesting that the alkyl surface modification does not result in a significant decrease in surface area of the SiO_2_ aerogel. This is consistent with our hypothesis that the short chain -O-Si-(CH_3_)_3_ occupies less space compared to the long chain counterparts. Both SiO_2_ aerogel and M-SiO_2_ aerogel show wide range pore size distribution from 2–80 nm and most of them are in the range of 2–20 nm, as shown in Figure 4b. After modifying with -O-S-(CH_3_)_3_, the average pore size slightly decreases since the surface of the M-SiO_2_ aerogel is connected with-O-Si-(CH_3_)_3_.

Leakage test was carried out to evaluate the RT60 encapsulating capacity of SiO_2_ aerogel and M-SiO_2_ aerogel, as shown in Figure S2. The RT60/SiO_2_ aerogel and RT60/M-SiO_2_ aerogel powders were placed on the paper and heated at 90 °C for 2 h. After cooling down to room temperature, the appearance of the RT60/SiO_2_ and RT60/M-SiO_2_ composites on the paper was recorded. The RT60/SiO_2_ aerogel shows no leakage with RT60 mass fraction of 55% and 60%. When the mass fraction of RT60 increased to 65%, obvious leakage was observed. As a contrast, no leakage was observed for the RT60/M-SiO_2_ aerogel with RT60 mass fraction of 75% and 80%. As the mass fraction of RT60 increased to 85%, the RT60/M-SiO_2_ aerogel started to leak. Obviously, the M-SiO_2_ aerogel demonstrated higher RT60 absorption capacity than SiO_2_, and the RT60/M-SiO_2_ exhibited better thermal stability than the RT60/SiO_2_ even, at a higher loading of RT60.

Figure 4a illustrates the mass fraction of RT60 absorbed in the SiO_2_ aerogel, unactivated M-SiO_2_ aerogel and activated M-SiO_2_ aerogel with different adsorption time. The encapsulation ratio of RT60 in SiO_2_ aerogel and M-SiO_2_ aerogel was calculated from the DSC results (vide infra). For SiO_2_ aerogel, the mass fraction rapidly increased from 0 to 50% in 20 min, and gently increased from 50.0% to 55.1% from 20–40 min. Then, the RT60 tended to a stationary mass ratio of 57.9%, which was considered as the maximum adsorption ratio of the SiO_2_ aerogel. For unactivated M-SiO_2_ aerogel, the mass fraction of RT60 increased to 65% in 19 min and tended to a stationary mass ratio of 70.5%. For activated M-SiO_2_ aerogel, the mass fraction of RT60 increased from 0 to 72.8% in 18 min, and slightly increased from 72.8% to 76.8% from 18–30 min. Finally, the RT60 in RT60/activated M-SiO_2_ aerogel reached a stationary mass ratio up to 80.0%. Above all, the activated M-SiO_2_ aerogel showed larger absorption capacity of RT60 than SiO_2_ aerogel and unactivated M-SiO_2_ aerogel. For comparison, the absorption capacities of previously reported porous oxides were summarized in Figure 4d and Table S1. The state of art montmorillonite [49], diatomite [50], expanded perlite [51] and SiO_2_ aerogel [40] could absorb paraffin at a mass ratio of 39.9%, 47.4%, 26.6%, and 54.8%. When modified with organic groups, the absorption capacity of the aforementioned porous oxides increased to 58.1% [52], 61% [53], 45.7%, and 69.5%. Obviously, our M-SiO_2_ aerogel showed the highest absorption capacity of up to 80%. We note that, in general, the absorbability of porous materials decreases with the length of the carbon chain of paraffin [54]. Therefore, in principle, the organic PCM we choose (RT60) to test the absorption ability of our M-SiO_2_ should be more difficult to encapsulate since it has the longest carbon chain among all the paraffins summarized in Figure 4d and Table S1. Therefore, the high RT60 encapsulation capacity of our M-SiO_2_ highlights its outstanding absorbability. As mentioned previously, the short chain-O-Si-(CH_3_)_3_ occupy less space compared to the long chain counterparts, and therefore does not result in significant shrinking in pore size. This is also accompanied with an additional benefit, the M-SiO_2_ aerogel with the short chain does not block (or block less) the pores of the SiO_2_ aerogel. Therefore, the less obstructed infiltration path allows for easier RT60 siphoning and consequently a higher absorption rate.

### 3.4. Morphology of SiO_2_, M-SiO_2_ Aerogels and Their PCMs Composites

**Figure 5:**
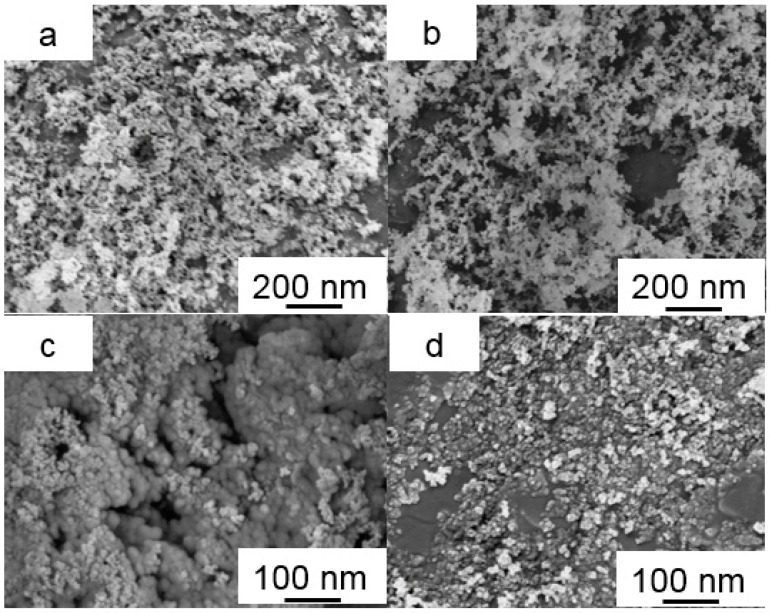
SEM images of SiO_2_ aerogel (**a**) and M-SiO_2_ aerogel (**b**), SiO_2_ aerogel-based phase change materials (PCMs) (**c**) and M-SiO_2_ aerogel-based PCMs (**d**).

**Figure 6:**
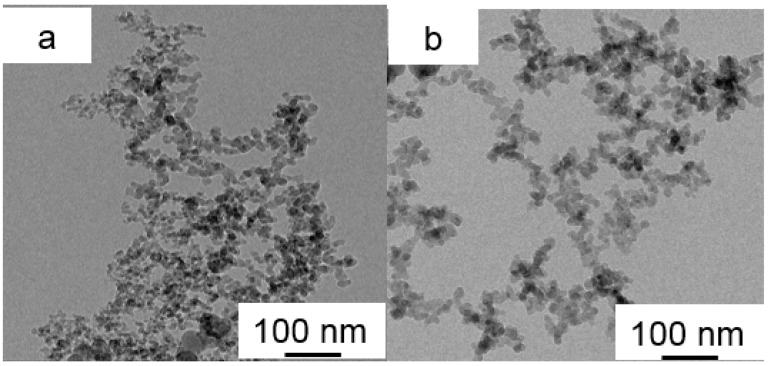
TEM images of SiO_2_ aerogel (**a**) and M-SiO_2_ aerogel (**b**).

The morphology and microstructure of SiO_2_ aerogel and M-SiO_2_ aerogel are shown in Figure 5. The SiO_2_ aerogel was created by the second accumulation of SiO_2_ particles with diameters of ~10 nm, the SiO_2_ particle built mesoporous mountain range and the pores were typical scaffold with SiO_2_ particles [55], as shown in Figure 5a. After modification, the surface of the M-SiO_2_ aerogel turned hydrophobic as illustrated previously in Figure 3b. Obviously, the short chain modification did not result in a significant narrowing of the pore size of the SiO_2_ aerogel, the M-SiO_2_ aerogel largely maintained the secondary pores of the SiO_2_ aerogel, as shown in Figure 5b. After modifying the surface with alkyl, the surface tension of SiO_2_ aerogel also increased. TEM analysis was carried out to further investigate the microstructure of SiO_2_ aerogel and M-SiO_2_ aerogel, as shown in Figure 6a,d. It could be clearly seen that the SiO_2_ particles of SiO_2_ aerogel accumulate together with a high surface area. When modified with -Si-(CH_3_)_3_, the M-SiO_2_ aerogel was dispersed better than SiO_2_ aerogel, the number of accumulated SiO_2_ particles decreases largely, verifying the successful surface modification of the SiO_2_ aerogel, as shown in Figure 6b. The SiO_2_ aerogel and M-SiO_2_ aerogel were used to absorb the RT60, and the morphology and microstructure of RT60/SiO_2_ aerogel and RT60/M-SiO_2_ aerogel are shown in Figure 5c,d. When absorbing RT60 into the pores of SiO_2_ aerogel, each SiO_2_ particle was inflated by RT60 like tomatoes on sticks, as shown in Figure 5c. The pores of SiO_2_ aerogel were only partially filled with RT60, since the SiO_2_ aerogel was hydrophilic. For comparison, the narrow space of the M-SiO_2_ aerogel was almost completely filled by RT60, which was attributed to hydrophobic nature of both the M-SiO_2_ aerogel and RT60.

### 3.5. Thermal Properties of RT60/SiO_2_ and RT60/M-SiO_2_ Aerogels

**Figure 7:**
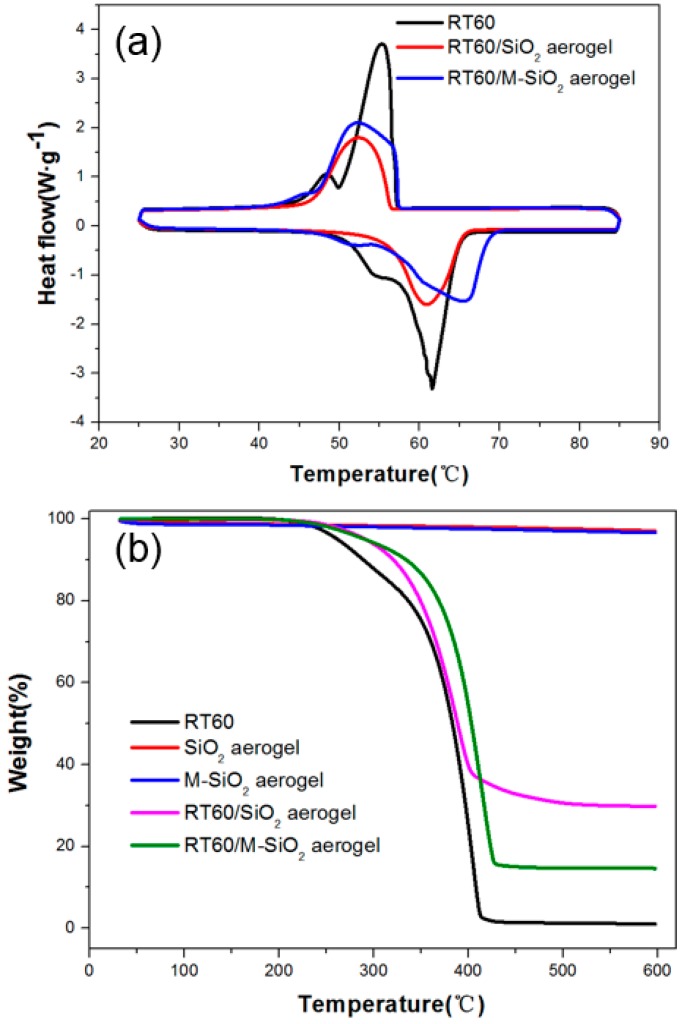
DSC curves of RT60, RT60/SiO_2_, RT60/M-SiO_2_ aerogels at a heating rate of 10 °C·min^−1^ (**a**), thermal stability measurements of SiO_2_ aerogel, M-SiO_2_, RT60, RT60/SiO_2_, RT60/M-SiO_2_ aerogels at 10 °C·min^−1^ (**b**).

The phase change temperatures and latent heats of RT60, RT60/SiO_2_ aerogel, and RT60/M-SiO_2_ aerogel were investigated. Figure 7a and Table S2 show the melting and freezing curves of RT60, RT60/SiO_2_ aerogel, and RT60/M-SiO_2_ aerogel. For the pure RT60, there was an endothermic peak in the melting DSC curve and an exothermic peak in the solidifying DSC curve. The melting and freezing temperatures were measured to be 57.98 and 56.61 °C for the pure RT60, 57.78 and 57.56 °C for RT60/SiO_2_ aerogel and 57.32 and 57.16 °C for RT60/M-SiO_2_ aerogel. The melting and freezing latent heats were measured to be 225.3 and 223.9 J·g^−1^ for the pure RT60, 130.5 and 129.4 J·g^−1^ for the pure RT60/SiO_2_ aerogel, and 180.2 and 178.9 J·g^−1^ for RT60/M-SiO_2_ aerogel. The phase change characteristics of the RT60/M-SiO_2_ aerogel were similar to those of the pure RT60, because there was no chemical reaction between RT60 and M-SiO_2_ aerogel in the preparation process. The encapsulation ratio (R) of RT60 by SiO_2_ aerogel and M-SiO_2_ aerogel was calculated from the DSC results using Equation (1).

(1) $$R=\frac{\Delta H_{m,Composite}}{\Delta H_{m,Paraffin}}\times 100\%$$

where Δ*H_m_*_,*Composite*_ and Δ*H_m,Paraffin_* represent the melting latent heat of RT60/SiO_2_ aerogel, RT60/M-SiO_2_ aerogel and RT60, respectively. The encapsulation ratio (*R*) of paraffin in the RT60/SiO_2_ aerogel and RT60/M-SiO_2_ aerogel was calculated to be 57.9 and 80.0%, the encapsulation ratio of M-SiO_2_ aerogel increased by 38.1% as compared to SiO_2_ aerogel. The encapsulation ratio of M-SiO_2_ aerogel was also higher than other porous oxides such as bentonite [29], diatomite [30], and expanded perlite [31], which showed encapsulation ratio of paraffin lower than 60% as previously summarized in Table S1.

The thermal stability of RT60, RT60/SiO_2_ aerogel and RT60/M-SiO_2_ aerogel were tested by TGA and the weight loss as a function of temperature is depicted in Figure 7b and Figure S5. The RT60 started to evaporate at about 230 °C, and the final weight loss percentage was nearly 100% at 410 °C. For SiO_2_ aerogel and M-SiO_2_ aerogel, there was no obvious weight loss till 600 °C, as confirmed in Figure S5. The SiO_2_ aerogel showed slight weight lost from 100% to 97.5% as the temperature rises from room temperature to 600 °C, the M-SiO_2_ aerogel had a weight loss stage of 1.5% at about 100 °C, which was attributed to the evaporation of the short chain alkyl. In the TGA curve of the RT60/SiO_2_ aerogel, the composite started to lose weight at ~270 °C and the final weight loss percentage was nearly 40%. The weight percentage of the RT60 in RT60/SiO_2_ aerogel was ~60%. The RT60/M-SiO_2_ composite started to lose weight at ~270 °C and the final weight loss percentage was nearly 20%. The weight percentage of the RT60 in RT60/M-SiO_2_ aerogel was ~80%, which matches well with the calculation from the DSC results. The TGA results also suggested that the PCM composite could slightly enhance the thermal stability of the RT60.

### 3.6. Reversible Stability of RT60/SiO_2_ and RT60/M-SiO_2_ Aerogels

**Figure 8:**
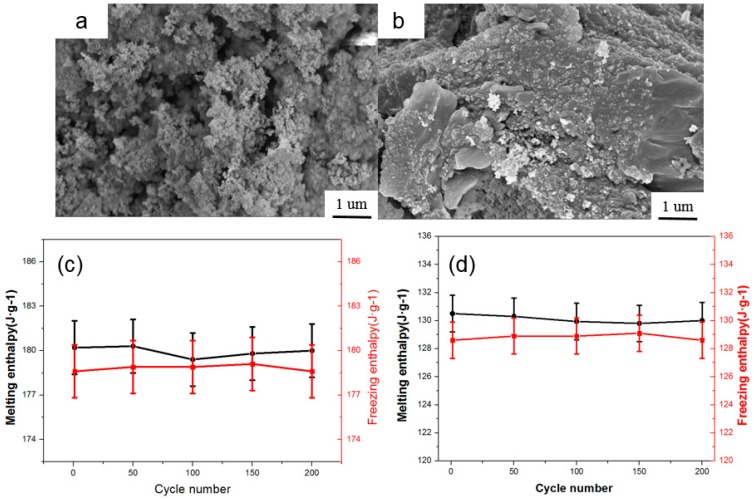
SEM image of RT60/SiO_2_ and RT60/M-SiO_2_ aerogels after 200 heating/cooling cycles (**a**,**b**). Absolute values of the melting and freezing enthalpy of RT60/SiO_2_ and RT60/M-SiO_2_ aerogels after different heating/cooling cycles (**c**,**d**) are shown.

To investigate the reversible stability, the RT60/M-SiO_2_ aerogel was heated and cooled with different heating-cooling cycles. As shown in Figure 8a,b, no obvious change was found in the morphology and microstructure of RT60/SiO_2_ aerogel and RT60/M-SiO_2_ aerogel after 200 heating/cooling cycles, meaning that the RT60/SiO_2_ and RT60/M-SiO_2_ aerogels have great reversible stability. To further verify the stability of RT60/SiO_2_ aerogel and RT60/M-SiO_2_ aerogel, the melting and freezing enthalpy of RT60/SiO_2_ aerogel and RT60/M-SiO_2_ aerogel after every heating/cooling cycles were recorded. The melting enthalpy of RT60/SiO_2_ aerogel and RT60/M-SiO_2_ aerogel maintained at 130.5 and 180.2 J·g^−1^ from 1–200 heating/cooling cycles. The freezing enthalpy of RT60/SiO_2_ aerogel and RT60/M-SiO_2_ aerogel kept at 128.8 and 178.8 J·g^−1^ in the heating/cooling process. These results confirmed the excellent reversible stability of RT60/SiO_2_ and RT60/M-SiO_2_ aerogels.

### 3.7. Thermal Conductivity of RT60/SiO_2_ and RT60/M-SiO_2_ Aerogels

**Figure 9:**
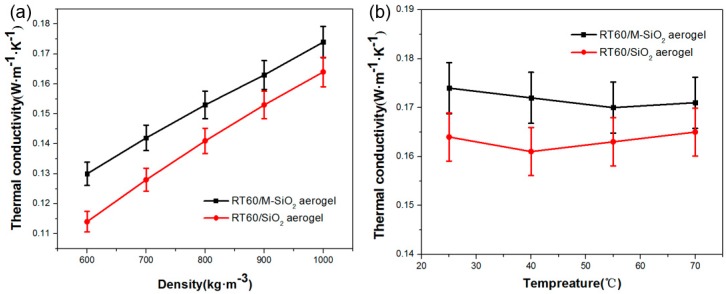
Thermal conductivity of RT60/SiO_2_ and RT60/M-SiO_2_ aerogels with different packing densities (**a**) and different temperatures (**b**).

The thermal conductivity of RT60/SiO_2_ and RT60/M-SiO_2_ aerogel blocks were measured and the results are shown in Figure 9. The thermal conductivity of RT60/SiO_2_ aerogel block increased from 0.114 to 0.164 W·m^−1^·K^−1^ as the packing density increases from 600 to 1000 kg·m^−3^, while the thermal conductivity of RT60/M-SiO_2_ aerogel block increased from 0.132 to 0.174 W·m^−1^·K^−1^, as shown in Figure 9a. The thermal conductivity of RT60/SiO_2_ aerogel and RT60/M-SiO_2_ aerogel kept at ~0.164 W·m^−1^·K^−1^ and 0.174 W·m^−1^·K^−1^ as the temperature increase from room temperature to 70 °C, as shown in Figure 9b. For comparison, the thermal conductivity of RT60 was measured to be 0.23 W·m^−1^·K^−1^. Note that the RT60/SiO_2_ aerogel and RT60/M-SiO_2_ aerogel block showed lower thermal conductivity than RT60, which is due to the low thermal conductivity of SiO_2_ aerogel and M-SiO_2_ aerogel (0.026 W·m^−1^·K^−1^) [56].

### 3.8. Thermal Capacity Behavior of RT60/SiO_2_ and RT60/M-SiO_2_ Aerogels

**Figure 10:**
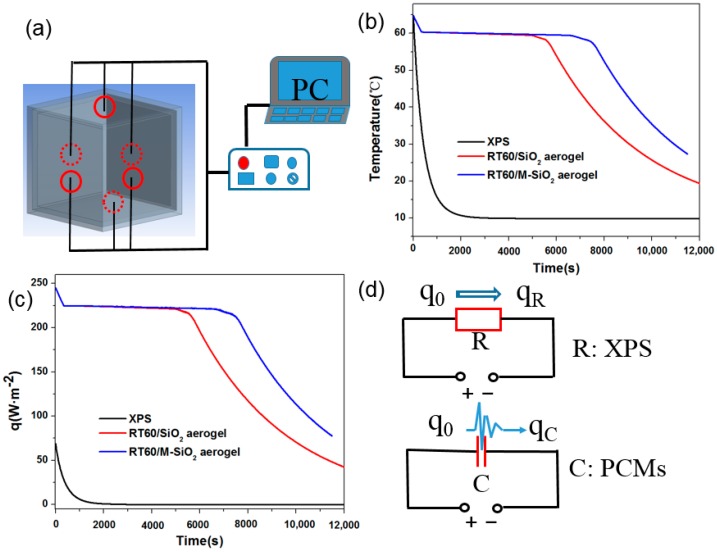
Experimental setup of thermal barrier property of M-SiO_2_ aerogel-based PCM (**a**), temperature response curve of SiO_2_ and M-SiO_2_ aerogels based PCMs (**b**), the rectified heat flow of the XPS form, SiO_2_ aerogel, and M-SiO_2_ aerogel-based PCMs (**c**), and the equivalent circuits of two kinds of thermal barrier boxes (**d**).

To investigate the thermal capacity behavior of SiO_2_ and M-SiO_2_ aerogel-based PCMs, the SiO_2_ and M-SiO_2_ aerogel-based PCMs were compressed into a cuboid with packing density of 1000 kg·m^−3^, and the cuboid was placed inner the XPS (polystyrene) box, as shown in Figure 10a. To record the temperature of SiO_2_ aerogel and M-SiO_2_ aerogel-based PCMs, the thermocouples were placed in the center of the PCMs cuboid with a thickness of 2 cm, the relative error of temperature data is within ± 1%. For comparison, the thermal insulation property of the XPS form with a thickness of 2 cm was also tested, as shown in Figure 9b. The XPS form, SiO_2_ aerogel and M-SiO_2_ aerogel-based PCMs decorative box was placed in the thermal oven to be heated at 65 °C. Then the box was then placed at an ambient temperature of 20 °C to perform the cooling energy storage property. Figure 10b showed that the temperature of XPS form decreased quickly from 65 to 30 °C in 400 s, while the temperature of SiO_2_ aerogel kept at 60 °C for 4800 s. The temperature of M-SiO_2_ aerogel-based PCMs decreased quickly from 65 to 60 °C in 700 s, and leveled at 60 °C from 700 to 8000 s, then dropped from 60 to 30 °C in 3000 s. The SiO_2_ aerogel and M-SiO_2_ aerogel-based PCMs demonstrated much better thermal insulation property than XPS form. The M-SiO_2_ aerogel-based PCMs could keep the temperature at 60 °C 2500 s longer than SiO_2_ aerogel-based PCMs. This is due to the large enthalpy of M-SiO_2_ aerogel-based PCMs.

To quantitatively evaluate the thermal capacity of SiO_2_ aerogel and M-SiO_2_ aerogel-based PCMs, the rectified heat flow from ambient to the box was calculated by Equation (2):

(2) $$q=\lambda \frac{T-T_{0}}{\Delta x}$$

where *q* is the apparent rectified heat flow (W·m^−2^), λ is the thermal conductivity(W·m^−1^·K^−1^), T is the temperature of PCMs side, T_0_ is the ambient temperature, Δ*x* is the thickness of the PCMs.

The rectification of the heat flow by SiO_2_ aerogel and M-SiO_2_ aerogel-based PCMs is shown in Figure 10c. The rectified heat flow of XPS form was only 75 W·m^−2^ and dropped rapidly to 0 W·m^−2^ in 2000 s, while the rectified heat flow of the SiO_2_ aerogel and M-SiO_2_ aerogel-based PCM was as high as 245 W·m^−2^, which was much higher than that of the XPS form. This is because the PCMs could absorb a larger mass of the heat than the XPS form. The M-SiO_2_ aerogel-based PCMs could rectify the heat flow for a longer time than SiO_2_ aerogel-based PCMs since the M-SiO_2_ aerogel-based PCMs have larger enthalpy.

In thermal circuit systems, the thermal behavior could be analyzed by the equivalent circuit method [57,58]. The XPS form could restrict the heat flow in the thermal circuit, and the PCMs could rectify the high-power heat flow in the thermal circuit. For thermal protection, the rectified effect of the PCMs is much better than the restriction effect of the XPS form. The rectified heat flow of the M-SiO_2_ aerogel-based PCM was 226.7% higher than that of the XPS form. The rectified heat flow decreased with exposure time as the temperature difference became small. The XPS form-based box could be equivalent to a thermal resistance, while the PCMs could be regarded as a heat capacitor, as shown in Figure 10d. In the thermal flow circuit, the thermal resistance could reduce the heat flow from *q*_0_ to *q*_R_, while the PCMs could rectify large heat flow from *q*_0_ to *q*_C_. The M-SiO_2_ aerogel-based PCM has a large thermal capacity with excellent rectification capability of heat flow, which is promising in high heat power systems includes thermal insulation applications, such as medicine, transportation and hot food storage.

## 4. Conclusions

In this work, hydrophobic SiO_2_ aerogel was obtained by short chain alkyl modification through a simple 2 step method. The N-hexane activation followed by short-chain alkane silanization yielded hydrophobic M-SiO_2_. In contrast to conventional long-chain modification of SiO_2_, the short chain modification did not result in significant shrinking of the pore size. The M-SiO_2_ showed outstanding absorption capacity of RT60 at a mass ratio up to 80%, which is significantly higher than un-modified SiO_2_ (58%) and also other porous oxides. The phase change composite RT60/M-SiO_2_ aerogel showed a high phase change enthalpy of 180 J·g^−1^ and low thermal conductivity of 0.178 W·m^−1^·K^−1^. The RT60/M-SiO_2_ composite was able to rectify of cooling thermal energy of 245 W·m^−2^, and could be used as a thermal capacitor. Moreover, the RT60 and M-SiO_2_ exhibited great compatibility, and the thermal properties of the RT60/M-SiO_2_ composite could be well maintained after 200 heat/cooling cycles. The hydrophobic M-SiO_2_ shows great potential for encapsulating organic PCMs for thermal protection applications. Specifically, the RT60/M-SiO_2_ aerogel is well suited for thermal protection during hot food transfer.

## Supplementary Materials

The following are available online at https://www.mdpi.com/2079-4991/9/4/657/s1, Figure S1: FT-IR spectrum of all samples, Figure S2: leakage test of RT60/SiO_2_ aerogel and RT60/M-SiO_2_ aerogel, Figure S3: XRD patterns of the SiO_2_ aerogel and M-SiO_2_ aerogel, Figure S4: XRD patterns of all samples, Figure S5: Weight loss of SiO_2_ aerogel and M-SiO_2_ aerogel, Figure S6: Melting and freezing behavior of RT60/SiO_2_ aerogel with different heating/cooling, Figure S7: Melting and freezing behavior of RT60/M-SiO_2_ aerogel with different heating/cooling cycle, Table S1: The absorption capacity of different supporting materials, Table S2: The melting and freezing behavior of SiO_2_ aerogel and M-SiO_2_ aerogel based PCM.
