# Supplementary material for: Short-Chain Modified SiO2 with High Absorption of Organic PCM for Thermal Protection
Source: Nanomaterials (Basel). 2019 Apr 25;9(4):657. doi: 10.3390/nano9040657 (PMC6523198; doi:10.3390/nano9040657)
Supplement: Supplementary file 1 [file nanomaterials-09-00657-s001.pdf]

Short-Chain Modified SiO_2 with High Absorption of Organic PCM for Thermal Protection

Fuxian Wang [1], Shiyuan Gao [2,3], Jiachuan Pan [1], Xiaomei Li [2,3] and Jian Liu [1,2,3,*]

1 Guangdong Provincial Key Laboratory of Emergency Test for Dangerous Chemicals, Guangdong Institute of Analysis, Guangzhou 510070, China; wangfuxian@fenxi.com.cn (F.W.); panjiachuan@fenxi.com.cn (J.P.)
2 The Engineering Research Center of None-Food Biomass Efficient Pyrolysis and Utilization Technology of Guangdong Higher Education Institutes, Dongguan University of Technology, Dongguan 523808, China; gsy0113@126.com (S.G.); L13728356646@163.com (X.L.)
3 Guangdong Provincial Key Laboratory of Distributed Energy Systems, School of Chemical Engineering and Energy Technology, Dongguan University of Technology, Dongguan 523808, China
* Correspondence: liujian@dgut.edu.cn; Tel.: +86-0769-22861808

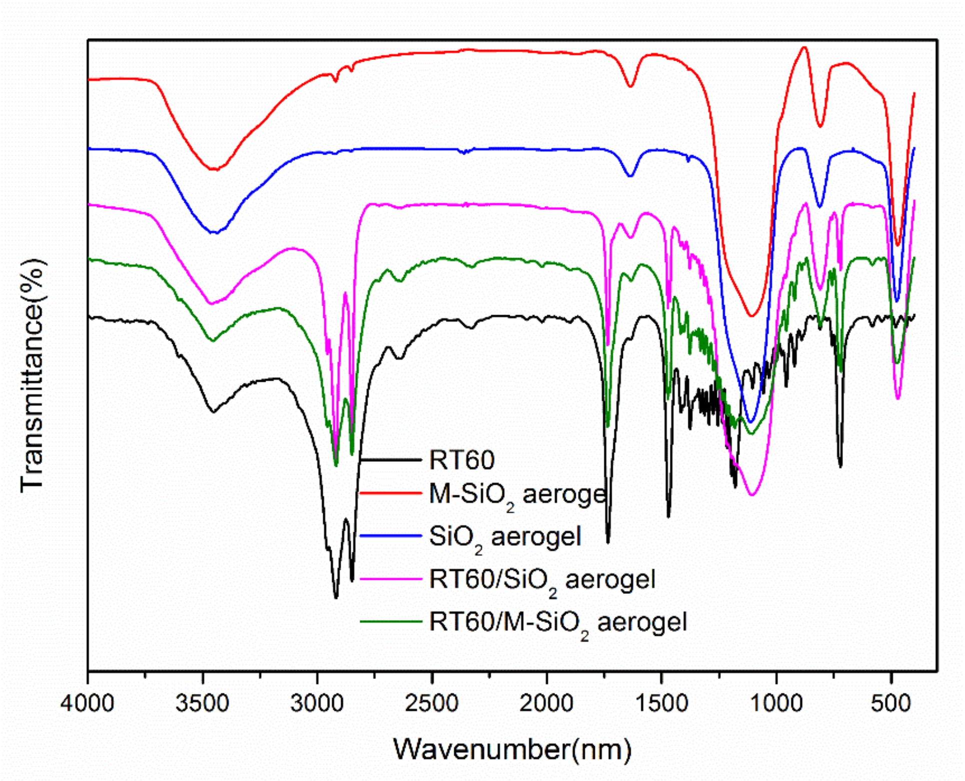

Figure S1. FT-IR spectrum of all samples.

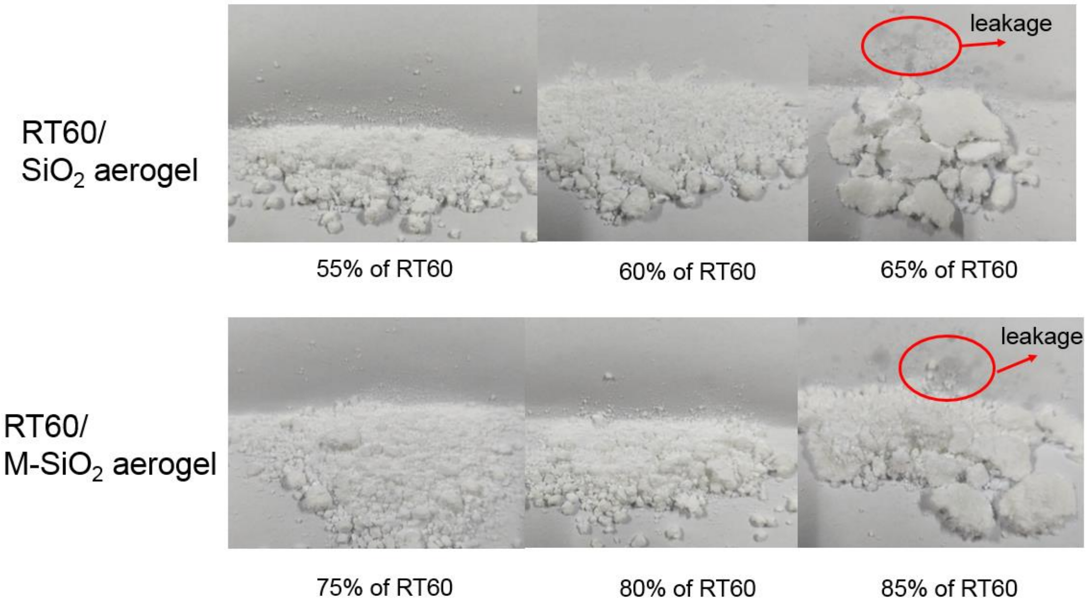

Figure S2. leakage test of RT60/SiO_2 aerogel and RT60/M-SiO_2 aerogel.

Table S1. The absorption capacity of different supporting materials.

Supporting materials	Modified agent	Organic PCMs	T_m/ ºC	$\triangle H_m/J{\cdot}g^{-1}$	Mass fraction %
Montmorillonite[1]	--	RT 20	20.8	53.6	39.9%
Modified montmorillonite[2]	Hexadecyltrimethyl ammonium bromide	RT 20	23.0	79.3	58.1%
Diatomite[3]	--	Paraffin	47.8	70.5	47.4%
Calcined diatomite[4]	--	Paraffin	57.3	125.9	61.0%
Expanded perlite[5]	--	Paraffin	17.2	35.5	26.6%
Modified expanded perlite[5]	--	Paraffin	16.3	60.9	45.7%
SiO_2 aerogel[6]	--	Paraffin	17.4	78.1	54.8%
Modified SiO_2 aerogel[6]	Dimethyldichlorosilance	Paraffin	17.7	98.9	69.5%
Our work	Hexamethyl disilazane	RT60	60	180.2	80.0%

The XRD results of SiO_2 aerogel and M-SiO_2 aerogel based PCM were shown in Figure S3 and S4. In the pattern of SiO_2 aerogel and M-SiO_2 aerogel, both samples has only one broad peak around 20°. The pattern of RT60 exhibits peaks at 10°, 14.2°, 21.5°, 23.9°, 38.9° and 42.1°. The sharp diffraction peaks at 21.5° and 23.9° are attributed to the diffractions of (110) and (200) crystal planes of paraffin[7]. Figure S4 shows the pattern of RT60/SiO_2 aerogel and RT60/M-SiO_2 aerogel, both RT60/SiO_2 aerogel and RT60/M-SiO_2 aerogel keep two sharp peaks at 21.5° and 23.9° of RT60, while other peaks strongly decrease the intensity, because the RT60 is encapsulated by SiO_2 aerogel and M-SiO_2 aerogel. In general, both of RT60/SiO_2 aerogel and RT60/M-SiO_2 aerogel do not exhibits new peaks in the XRD pattern, verifying the physical combination of RT60 and SiO_2 aerogel/M-SiO_2 aerogel.

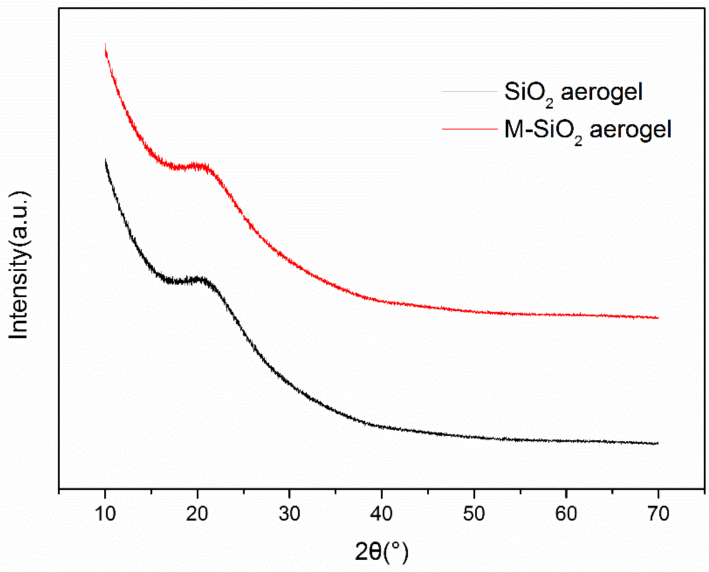

Figure S3. XRD patterns of the SiO_2 aerogel and M-SiO_2 aerogel.

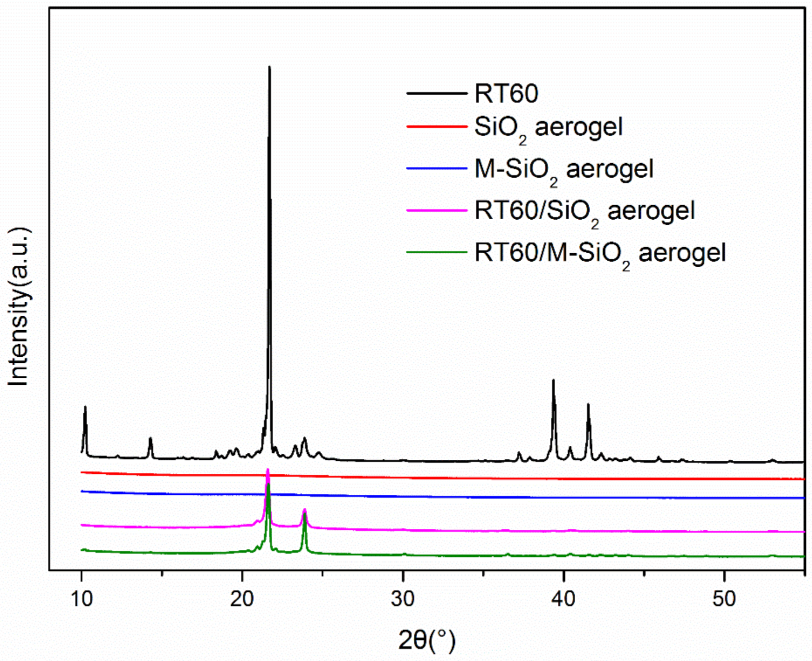

Figure S4. XRD patterns of all samples.

Table S2. The melting and freezing behavior of SiO_2 aerogel and M-SiO_2 aerogel based PCM.

	T_m/ ºC	ΔH_m/J·g^{-1}	T_f/ ºC	ΔH_f/J·g^{-1}	η/%
RT60	57.98	225.3	56.61	223.6	-
RT60/SiO_2 aerogel	57.78	130.5	57.56	129.4	57.9
RT60/M-SiO_2 aerogel	57.32	180.2	57.16	178.9	80.0

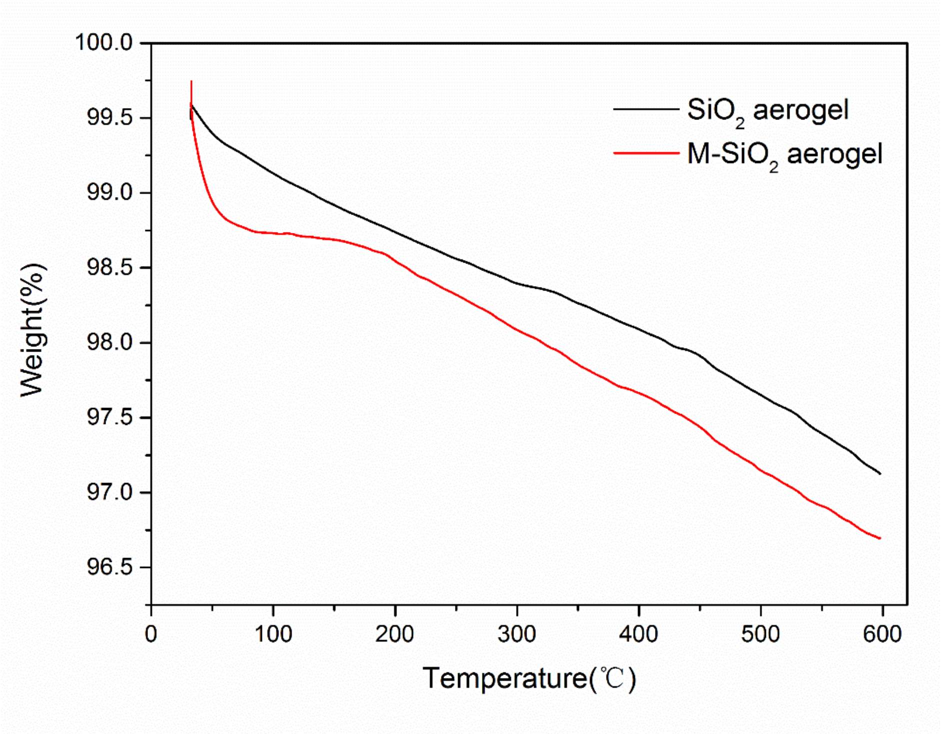

Figure S5. Weight loss of SiO_2 aerogel and M-SiO_2 aerogel.

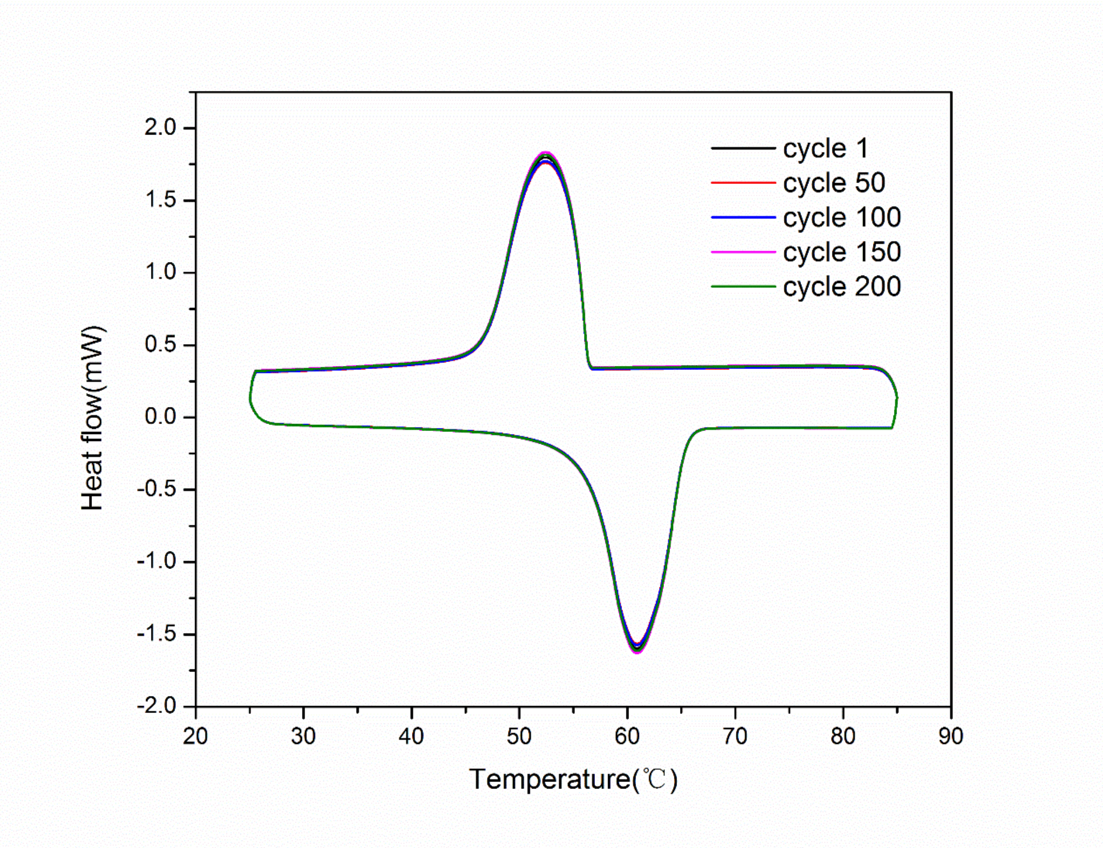

Figure S6. Melting and freezing behavior of RT60/ SiO_2 aerogel with different heating/cooling. cycle.

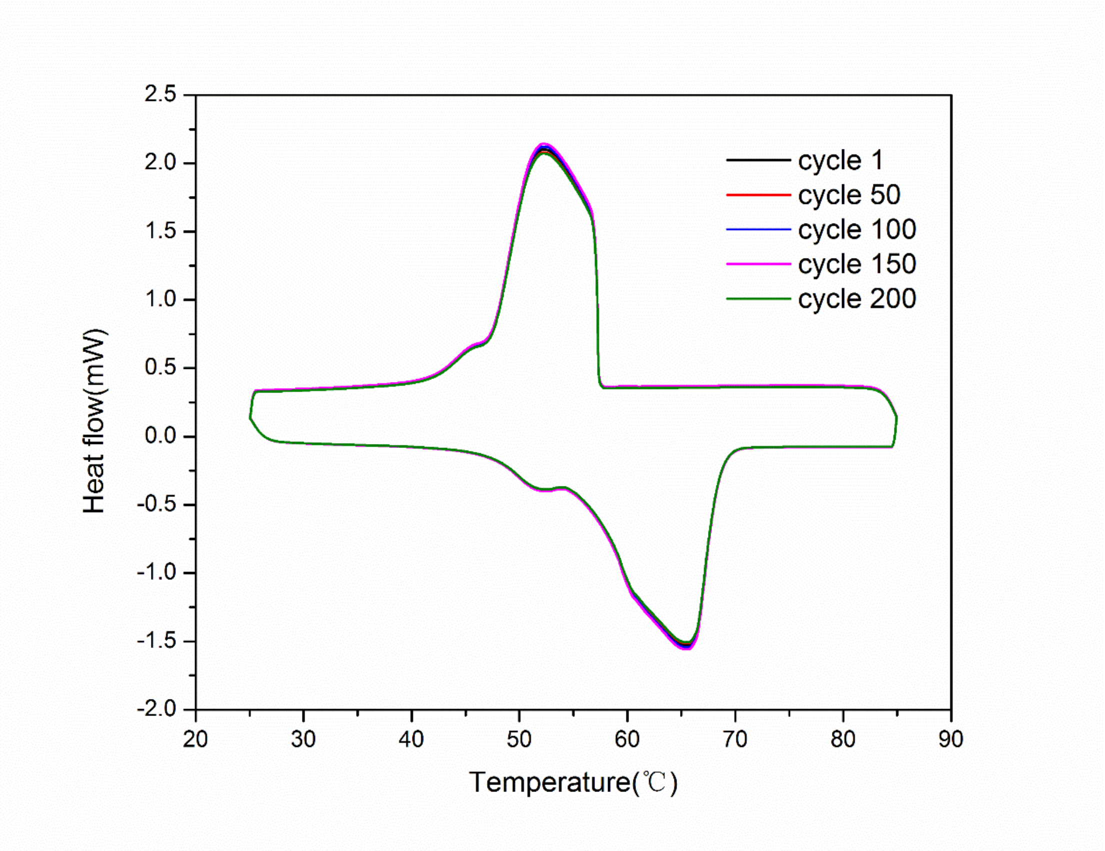

Figure S7. Melting and freezing behavior of RT60/M-SiO_2 aerogel with different heating/cooling cycle.

REFERENCE

1. Fang, X.; Zhang, Z. A novel montmorillonite-based composite phase change material and its applications in thermal storage building materials. Energy and Buildings 2006, 38, 377-380.

2. Fang, X.; Zhang, Z.; Chen, Z. Study on preparation of montmorillonite-based composite phase change materials and their applications in thermal storage building materials. Energy Conversion and Management 2008, 49, 718-723.

3. Xu, B.; Li, Z. Paraffin/diatomite composite phase change material incorporated cement-based composite for thermal energy storage. Applied Energy 2013, 105, 229-237.

4. Sun, Z.; Zhang, Y.; Zheng, S.; Park, Y.; Frost, R.L. Preparation and thermal energy storage properties of paraffin/calcined diatomite composites as form-stable phase change materials. Thermochimica Acta 2013, 558, 16-21.

5. Ramakrishnan, S.; Sanjayan, J.; Wang, X.; Alam, M.; Wilson, J. A novel paraffin/expanded perlite composite phase change material for prevention of pcm leakage in cementitious composites. Applied Energy 2015, 157, 85-94.

6. Li, H.; Chen, H.; Li, X.; Sanjayan, J.G. Development of thermal energy storage composites and prevention of pcm leakage. Applied Energy 2014, 135, 225-233.

7. Zhang, Z.; Zhang, N.; Peng, J.; Fang, X.; Gao, X.; Fang, Y. Preparation and thermal energy storage properties of paraffin/expanded graphite composite phase change material. Applied Energy 2012, 91, 426-431.
